# Pathology

**DOI:** 10.1080/13577140120097094

**Published:** 2001-11


### PATHOLOGY

### 023 Expression of HER2/Neu (HER2) and EGFR In Osteosarcoma (Os)

James C Wittig[1], Lorna Cruz[3], Felasfa Wodajo[1], WM Chu[2], Kristen L Kellar-Graney[1], Nita Seibel[4], Dhruv Kumar[3], Martin M Malawer[1], Dennis A Priebat[2]
*[1]Dept of Orthopedic Oncology Washington Cancer Institute Washington Hospital Center, [2]Deptartment of Hematology/Oncology Washington Cancer Institute Washington Hospital Center, [3]Department of Pathology Washington Hospital Center, [4]Children's National Medical Center Department of Pediatric Hematology/Oncology*

*Objective:* Overexpression of protooncogenes and cellular growth factor receptors have been implicated in the development of osteosarcomas. HER2 (human epidermal growth factor receptor 2) is a 185-Kd transmembrane glycoprotein receptor that is the product of the c-erbB-2 protooncogene. It is structurally homologous to EGFR (epidermal growth factor receptor). Recent studies have reported that 42%-45% of OS overexpress HER2 and that overexpression of HER2 in OS correlates with a worse event-free survival, and a poor histologic response to induction chemotherapy. This has stimulated interest in investigating the use of anti-HER2 monoclonal antibodies in treatment protocols as part of initial or salvage chemotherapy regimens for poor prognosis patients. Expression of EGFR in OS has not been thoroughly investigated although its overexpression by other tumor types has been correlated with a poor prognosis. The purpose of this study was to examine the expression of HER2 and EGFR in OS and to determine the relationship between overexpression and survival, response to induction chemotherapy, local and systemic recurrence, and various other potential prognostic factors.
*Methods:* Archival paraffin embedded tissue from 32 patients with the diagnosis of high-grade OS were stained for HER2 (Hercep Test- DAKO Corp) and EGFR (Mouse Anti-EGFR, ZYMED LABS) via standard immunohistochemical techniques. Cellular membrane staining was graded 0 to 3+ according to intensity. The receptors were considered to be overexpressed if grade 2 or 3 staining was present.
*Results:* HER2 was not expressed in any specimen. (All specimens demonstrated grade 0 staining for HER2.) EGFR was overexpressed in five (16%) specimens. Overexpression of EGFR did not correlate with prognosis or with any significant prognostic variables.
*Conclusion:* HER2 and EGFR do not demonstrate significant nor consistent overexpression by OS cells. They are unlikely to represent important prognostic variables. Therapy with anti-HER2 antibody or IMC-C225 (anti-EGFR ab) may not be beneficial. Further investigation is required.

### 027 HER2/ERB2 Expression in Synovial Sarcoma

Dafydd Gareth Thomas[1], Donita L Sanders[1], J Sybil Biermann[2], Laurence Baker[3], Thomas Giodano[1]
*[1]Department of Pathology University of Michigan Medical School, [2]Department of Orthopedic Sugery University of Michigan Hospitals, [3]Department of Internal Medicine University of Michigan Hospitals*

*Objective:* Synovial sarcoma is a high-grade soft tissue sarcoma characterized by a bi-phasic morphology and is associated with a poor prognosis. Most synovial sarcoma posses a specific chromosomal translocation (t(X:18) p11.2;q11.2). Surgery and adjuvant chemotherapy has improved the outcome of local disease, however, management of distant metastasis remains problematic. Accordingly there is a need for alternate adjuvant therapies. The c-erbB2 proto-oncogene encodes the human epidermal growth factor receptor 2 (HER-2/neu), a membrane bound tyrosine kinase, overexpressed in a number of carcinomas.
*Methods:* Archival cases of synovial sarcomas (n=16) were assessed for HER-2/neu oncogene expression by standard immunohistochemical techniques.
*Results:* Eleven of the cases (68%) demonstrated the membranous staining reminiscent of over expression in breast carcinomas. To validate the immunohistochemistry results, RT-PCR using archival material was performed and demonstrated the presence of mRNA for c-erbB2 in 10 of 13 (72%) of cases.
*Conclusion:* These results demonstrate c-erbB2 expression in the majority of synovial sarcoma and suggest treatment with recombinant humanized anti-HER2 monoclonal antibodies may represent an appropriate alternate therapy for patients who have failed conventional therapy.

### 044 Diagnostic Impact of Histologic Parameters in Differentiating Enchondroma from Grade I Central Chondrosarcoma

D Eefting[1], M J Geirnaerdt[2], S Le Cessie[3], A H Taminiau[4], P C Hogendoorn[1]
*[1]Department of Pathology, Leiden University Medical Center, [2]Department of Radiology, Leiden University Medical Center, [3]Department of Medical Statistics, Leiden University Medical Center, [4]Department of Orthopedic Surgery, Leiden University Medical Center*

*Objective:* Because of the difference in clinical management of enchondromas and central grade I chondrosarcomas it is important to make a correct preoperative diagnosis. The purpose of this study was to investigate the diagnostic value of histologic parameters in differentiating enchondroma and central grade I chondrosarcoma.
*Methods:* Cytologic and tissue-architectural features of 57 patients, with 20 enchondromas and 37 central grade I chondrosarcomas, were assessed. During the microscopically assessment, the observer was aware of the localization of the tumor, the age of the patient and clinical symptoms (like swelling, pain or fracture). A final diagnosis was used as gold standard obtained after at least 10 years of follow-up and assessed in a multidisciplinary meeting with full clinical, radiological and pathology data available. Tumors of the digits and toes were excluded because of their specific (benign) clinical behavior, despite aggressive morphologic features. The chi-square test, classification trees and logistic regression were used to assess the discriminating power of the individual parameters and to find an optimal set of parameters.
*Results:* Entrapment, cellularity, nuclear polymorphism and distribution of cells were the individual parameters with the most discriminating strength ($p<0.001$). The combination of high cellularity, present host bone entrapment, open chromatin, mucoid matrix quality >= 20% and an age above 45 years gives a perfect prediction. Also, the matrix quality proved to be a strong discriminating feature. A statistical classification tree shows that if mucoid matrix degeneration is more then 20% or entrapment is present (or both of them), the tumor will be almost certain malignant and otherwise almost certain benign.
*Conclusion:* The application of a combination of 5 histologic parameters showed a optimal ability to differentiate between enchondromas and central grade I chondrosarcomas. With a simple classification tree, based on 2 parameters, 54 of the 57 (94.7%) cases were assessed correct (sensitivity 95% and specificity 95%). Using this diagnostic approach combined with an tailored radiological assessment will lead to an optimal therapeutic classification.

DOI: 10.1080/13577140120097094

**046 Tissue-Array for Immunohistochemical Studies in Soft Tissue Sarcoma**

Jacob Engellau[1], Måns Åkerman[2], Harald Anderson[3], Henryk A Domanski[2], Thor A Alvegård[1], Mef C Nilbert[1]
*[1]Dept. of Oncology, University hospital, [2]Dept. of Pathology, University hospital, [3]Dept. of Cancer Epidemiology, University hospital*

*Objective:* Malignant fibrous histiocytoma (MFH) represents a heterogeneous soft tissue sarcoma entity. We have compared different methods to determine immunohistochemical (ICH) staining in whole-tissue sections, evaluated the tissue-array technique and assessed ICH heterogeneity using the proliferation marker Ki-67.
*Methods:* The techniques were evaluated in 47 tumor blocks from 11 MFH. Whole-tissue sections were assessed using two reference methods (counting 400 cells along a line or counting 10 high power fields, x40). Tissue-array utilized multiple 0.6 mm tumor biopsies.
*Results:* The whole-tissue methods gave Ki-67 expression levels of 13% and 11%, respectively and the tissue-array technique correlated well with the whole-tissue ICH with average 8.6% higher Ki-67 expression in the array-sections. ICH heterogeneity gave a median standard deviation (SD) of 2.3% within the tumor blocks and of 2.5% between the blocks from the same tumor.
*Conclusion:* We conclude that the tissue-array method yields good quality ICH staining and expression levels for Ki-67 comparable to whole-tissue staining in MFH. Because of tumor heterogeneity several tumor blocks should ideally be studied and due to loss of biopsies in the array-process multiple biopsies should be taken. The feasibility of tissue-array offers new possibilities for ICH studies using multiple markers in large tumor series.

**052 Autocrine and Paracrine Regulation of Metastatic Growth in Canine Osteosarcoma**

Dafydd G. Thomas[1], Kirsten M. Leu[2], Stephen Hewitt[3], Chand Khanna[3], Lee Helman[3], Thomas J. Giordano[1], Laurence Baker[2]
*[1]Department of Pathology University of Michigan Hospitals, [2]Division of Hematology & Oncology Department of Internal Medicine University of Michigan Hospitals, [3]Pediatric Oncology Branch NCI, NIH*

*Objective:* Canine osteosarcoma is a naturally occurring tumor homologous to their human tumors in morphology and behavior. Consequently the canine neoplasm is a useful model to test new disease modifying agents. Recently, the treatment of other mesenchymal tumors using anti-tyrosine kinase drugs, especially drugs targeted against the PDGF family has been proposed. The PDGF family consists of 3 dimeric proteins, which act as ligands for the 2 receptors (PDGFR-a and PDGFR-b) with tyrosine kinase activity. The dimers are formed by subunits A and B can bind as the heterodimer or the homodimers. PDGF-B is similar to proto-oncogene c-sis, which is involved in the malignant transformation of fibroblast cultures. The a receptor is stimulated by all three isoforms of the ligand, whereas the b-receptor only binds the PDGF-B homodimer with high affinity.
*Methods:* In this study, the expression of the PDGF family members in 84 canine osteosarcomas (73 primary and 11 pulmonary metastatic neoplasms) was examined using a tissue microarray and immunohistochemistry.
*Results:* PDGF-A and PDGF-B expression was detected in 43 (51%) and 8 tumors (9.5%) respectively. 66 tumors (78.5%) expressed PDGFR-a and 52 tumors (61.9%) expressed PDGFR-b respectively. Concomitant expression of the ligand (PDGF-A and PDGF-B) and receptor (PDGFR-a) was seen in all of the metastatic lesions, whereas primary neoplastic lesions demonstrated likewise concomitant expression in 27 lesions (36%).
*Conclusion:* These results suggest autocrine and paracrine stimulation of osteosarcoma cells in metastatic lesions. Furthermore, these results suggest metastatic osteosarcoma may be a possible target for anti-PDGFR drugs.

